# A kernel-based integration of genome-wide data for clinical decision support

**DOI:** 10.1186/gm39

**Published:** 2009-04-03

**Authors:** Anneleen Daemen, Olivier Gevaert, Fabian Ojeda, Annelies Debucquoy, Johan AK Suykens, Christine Sempoux, Jean-Pascal Machiels, Karin Haustermans, Bart De Moor

**Affiliations:** 1 Department of Electrical Engineering (ESAT-SCD), Katholieke Universiteit Leuven, Kasteelpark Arenberg, 3001 Leuven, Belgium; 2 Department of Experimental Radiotherapy, Katholieke Universiteit Leuven, UZ Herestraat, 3000 Leuven, Belgium; 3 Department of Pathology, Université Catholique de Louvain, St Luc University Hospital, Avenue Hippocrate, 1200 Brussels, Belgium; 4 Department of Medical Oncology, Université Catholique de Louvain, St Luc University Hospital, Avenue Hippocrate, 1200 Brussels, Belgium

## Abstract

**Background:**

Although microarray technology allows the investigation of the transcriptomic make-up of a tumor in one experiment, the transcriptome does not completely reflect the underlying biology due to alternative splicing, post-translational modifications, as well as the influence of pathological conditions (for example, cancer) on transcription and translation. This increases the importance of fusing more than one source of genome-wide data, such as the genome, transcriptome, proteome, and epigenome. The current increase in the amount of available omics data emphasizes the need for a methodological integration framework.

**Methods:**

We propose a kernel-based approach for clinical decision support in which many genome-wide data sources are combined. Integration occurs within the patient domain at the level of kernel matrices before building the classifier. As supervised classification algorithm, a weighted least squares support vector machine is used. We apply this framework to two cancer cases, namely, a rectal cancer data set containing microarray and proteomics data and a prostate cancer data set containing microarray and genomics data. For both cases, multiple outcomes are predicted.

**Results:**

For the rectal cancer outcomes, the highest leave-one-out (LOO) areas under the receiver operating characteristic curves (AUC) were obtained when combining microarray and proteomics data gathered during therapy and ranged from 0.927 to 0.987. For prostate cancer, all four outcomes had a better LOO AUC when combining microarray and genomics data, ranging from 0.786 for recurrence to 0.987 for metastasis.

**Conclusions:**

For both cancer sites the prediction of all outcomes improved when more than one genome-wide data set was considered. This suggests that integrating multiple genome-wide data sources increases the predictive performance of clinical decision support models. This emphasizes the need for comprehensive multi-modal data. We acknowledge that, in a first phase, this will substantially increase costs; however, this is a necessary investment to ultimately obtain cost-efficient models usable in patient tailored therapy.

## Background

Kernel methods are a powerful class of methods for pattern analysis. In recent years, they have become a standard tool in data analysis, computational statistics, and machine learning applications [1]. Based on a strong theoretical framework, their rapid uptake in applications such as bioinformatics [2], chemoinformatics, and even computational linguistics is due to their reliability, accuracy, and computational efficiency. In addition, they have the capability to handle a very wide range of data types (for example, kernel methods have been used to analyze sequences, vectors, networks, phylogenetic trees, and so on). The ability of kernel methods to deal with complex structured data makes them ideally positioned for heterogeneous data integration. More specifically, in this study we used a weighted least squares support vector machine (LS-SVM), an extension of the support vector machine (SVM) for supervised classification [3-5]. Compared to the SVM, the LS-SVM is easier and faster for high dimensional data because the quadratic programming problem is converted into a linear problem. To account for the unbalancedness in many two-class problems, this linear problem is extended with weights that are different for the positive and negative classes.

The growing amount of data combined with factors such as time, cost, and personalized treatment is complicating clinical decision making. Using advanced mathematical models such as the above mentioned LS-SVM can aid clinical decision support because information arising from clinical risk factors (for example, tumor size, number of positive lymph nodes) is not accurate enough to reliably predict patient prognoses. Patients with the same clinical and pathological characteristics but different clinical outcomes can potentially be discerned with microarray technology. This technology investigates the transcriptomic make-up of a tumor in one experiment. A decade ago, it was first used in cancer studies to classify tissues as cancerous or non-cancerous [6,7]. Within the domain of cancer, microarray technology has earned a prominent place for its capacity to characterize underlying tumor behavior in detail. Although the first gene expression profile signature is being validated in clinical trials [8-10], microarray technology can not measure the complete transcription profile due to the limited number of probes per gene on a chip; nor does the transcriptome completely reflect the biology underlying a disease.

Besides transcription, pathological conditions such as cancer also influence alternative splicing, chromosomal aberrations, and methylation [11,12]. For example, chromosomal aberrations have been found in the general population as well as in all major tumor types [13,14]. These regions of increased or decreased DNA copy number can be detected using, for example, array comparative genomic hybridization (CGH) technology. This technique measures copy number variations (CNVs) within the entire genome of a disease sample compared to a normal sample [11]. Many small aberrations have emerged as prognostic and predictive markers. Numerous aberrations, however, also affect large genomic regions, encompassing multiple genes or whole chromosome arms.

Due to differential splicing or post-translational modifications such as phosphorylation or acetylation, the proteome is many orders of magnitude bigger than the transcriptome. This makes the proteome, which reflects the functional state of the cell, a potentially richer source of data for unraveling diseases [15]. It can be measured using mass spectrometry [16], or protein or antibody microarrays [17]. Additionally, other available omics data, such as epigenomics - the study of epigenetic changes such as DNA methylation and histone modifications [12] - and single nucleotide polymorphism genotyping [18], should be considered as they promise to be useful in unraveling cancer mechanisms and the refinement of their molecular descriptions. Although the technologies are available, joint analysis of multiple hierarchical layers of biological regulation is at a preliminary stage.

In this study we investigate whether the integration of information from multiple layers of biological regulation improves the prediction of cancer outcome.

### Related work

Other research groups have already proposed the idea of data integration, but most groups have only investigated the integration of clinical and microarray data. Tibshirani and colleagues [19] proposed such a framework by reducing the microarray data to one variable, addable to models based on clinical characteristics such as age, grade, and size of the tumor. Nevins and colleagues [20] combined clinical risk factors with metagenes (that is, the weighted average expression of a group of genes) in a tree-based classification system. Wang *et al. *combined microarray data with knowledge on two clinicopathological variables by defining a gene signature only for the subset of patients for whom the clinicopathological variables were not sufficient to predict outcome [21].

A further evolution can be seen in studies in which two omics data sources are simultaneously considered, in most cases microarray data combined with proteomics or array CGH data. Much literature on such studies involving data integration already exists. However, the current definition of the integration of high-throughput data sources as it is used in the literature differs from our point of view.

In a first group of integration studies, heterogeneous data from different sources were analyzed sequentially; that is, one data source was analyzed while the second was used as confirmation of the found results or for further deepening the understanding of the results [22]. Such approaches are used for biological discovery and a better understanding of the development of a disease, but not for predictive purposes. For example, Fridlyand and colleagues [23] found three breast tumor subtypes with a distinct CNV pattern based on array CGH data. Microarray data were subsequently analyzed to identify the functional categories that characterized these subtypes. Tomioka *et al. *[24] analyzed microarray and array CGH data of patients with neuroblastoma in a similar way. Genomic signatures resulted from the array CGH data, while molecular signatures were found after the microarray analysis. The authors suggested that a combination of these independent prognostic indicators would be clinically useful.

The term data integration has also been used as a synonym for data merging in which different data sets are concatenated at the database level by cross-referencing the sequence identifiers, which requires semantic compatibility among data sets [25,26]. Data merging is a complex task due to, for example, the use of different identifiers, the absence of a 'one gene-one protein' relationship, alternative splicing, and measurement of multiple signals for one gene. In most studies, the concordance between the merged data sets and their interpretation in the context of biological pathways and regulatory mechanisms are investigated. Analyses of the merged data set by clustering or correlating the protein and microarray data can help identify candidate targets when changes in expression occur at both the gene and protein levels. However, there has been only modest success from correlation studies of gene and protein expression. Bitton *et al*. [27] combined proteomics data with exon array data, which allowed a much more fine-grained analysis by assigning peptides to their originating exons instead of mapping transcripts and proteins based on their IDs.

Our definition for the combination of heterogeneous biological data is different. We integrate multiple layers of experimental data into one mathematical model for the development of more homogeneous classifiers in clinical decision support. For this purpose, we present a kernel-based integration framework. Integration occurs within the patient domain at a level not so far described in the literature. Instead of merging data sets or analyzing them in turn, the variables from different omics data are treated equally. This leads to the selection of the most relevant features from all available data sources, which are combined in a machine learning-based model. We were inspired by the idea of Lanckriet and colleagues [28]. They presented an integration framework in which each data set is transformed into a kernel matrix. Integration occurs on this kernel level without referring back to the data. They applied their framework to amino acid sequence information, expression data, protein-protein interaction data, and other types of genomic information to solve a single classification problem: the classification of transmembrane versus non-transmembrane proteins. In this study by Lanckriet and colleagues, all considered data sets were publicly available. This requires a computationally intensive framework for determining the relevance of each data set by solving an optimization problem. Within our set-up, however, all data sources are derived from the patients themselves. This makes the gathering of these data sets highly costly and limits the number of data sets, but guarantees more relevance for the problem at hand.

We previously investigated whether the prediction of distant metastasis in breast cancer patients could be improved when considering microarray data besides clinical data [29]. In this manuscript, we consider not only microarray data but also high-throughput data from multiple biological levels. Three different strategies for clinical decision support are proposed: the use of individual data sets (referred to as step A); an integration of each data type over time by manually calculating the change in expression (step B); and an approach in which data sets are integrated over multiple layers in the genome (and over time) by treating variables from the different data sets equally (step C).

We apply our framework to two cases, summarized in Table 1. In the first case on rectal cancer, tumor regression grade, lymph node status, and circumferential margin involvement (CRM) are predicted for 36 patients based on microarray and proteomics data, gathered at two time points during therapy. The second case on prostate cancer involves microarray and copy number variation data from 55 patients. Tumor grade, stage, metastasis, and occurrence of recurrence were available for prediction [30,31].

**Table 1:** Overview of the two case studies on rectal and prostate cancer

	Data set I: rectal cancer	Data set II: prostate cancer
Number of samples	36	55
		
Data sources	Microarray	Microarray
	Proteomics	Genomics
		
Number of features (after preprocessing)	*T*_0_: 6,913 genes; 90 proteins	6,974 genes
	*T*_1_: 6,913 genes; 92 proteins	7,305 CNVs
		
Outcomes	WHEELER	GRADE
	pN-STAGE	STAGE
	CRM	METASTASIS
		RECURRENCE

## Materials and methods

### Data set I: rectal cancer

#### Patients and treatment

Forty patients with rectal cancer (T3-T4 and/or N+) from seven Belgian centers were enrolled in a phase I/II study investigating the combination of cetuximab, capecitabine, and external beam radiotherapy in the preoperative treatment of patients with rectal cancer [32]. These patients received preoperative radiotherapy (1.8 Gy, 5 days/week for 5 weeks) in combination with cetuximab (initial dose 400 mg/m^2 ^intravenous given 1 week before the beginning of radiation followed by 250 mg/m^2^/week for 5 weeks) and capecitabine for the duration of radiotherapy (first dose level, 650 mg/m^2^orally twice-daily; second dose level, 825 mg/m^2 ^twice-daily; including weekends). Details of the eligibility criteria, pretreatment evaluation, radiotherapy, chemotherapy and cetuximab administration, surgery, follow-up, and histopathological assessment of response to chemoradiation have been published [32].

#### Data preprocessing

Tissue and plasma samples were gathered at three time points: before treatment (*T*_0_); after the first loading dose of cetuximab but before the start of radiotherapy with capecitabine (*T*_1_); and at the moment of surgery (*T*_2_). All experimental procedures were done following standard laboratory procedures, or following the manufacturers' instructions. Because of the exclusion of some patients due to a missing outcome value, death before surgery, or not having surgery, the data set ultimately contained 36 patients.

The frozen tissue samples were hybridized to Affymetrix human U133 2.0 plus gene chip arrays. The resulting data were first preprocessed for each time point separately using robust multichip analysis [33]. Secondly, the number of features was reduced from 54,613 probe sets to 27,650 genes by taking the median of all probe sets that matched on the same gene. Probe sets that matched on multiple genes were excluded because of the danger of cross-hybridization. Taking into account the low signal-to-noise ratio of microarray data, we finally filtered out genes with low variation across all samples. Only retaining the genes with a variance in the top 25% reduced the number of features to 6,913 genes.

Ninety-six proteins known to be involved in cancer were measured in the plasma samples using a Luminex 100 instrument. Proteins that had absolute values above the detection limit in less than 20% of the samples were excluded for each time point separately. This resulted in the exclusion of six proteins at *T*_0_, four at *T*_1_, and six at *T*_2_. The proteomics expression values of transforming growth factor alpha, which had too many values below the detection limit, were replaced by the results of ELISA tests performed at the Department of Experimental Oncology in Leuven, Belgium. For the remaining proteins the missing values were replaced by half of the minimum detected for each protein over all samples, and values exceeding the upper limit were replaced by the upper limit value. Because most of the proteins had a positively skewed distribution, a log transformation (base 2) was performed.

In this paper, only the data sets at *T*_0 _and *T*_1 _were used because our goal is to predict the four different outcomes before therapy or early in therapy.

#### Response classification

A semiquantitative classification system has been described by Wheeler *et al. *[34] for determining histopathological tumor regression (that is, the therapy response). There are also two prognostic factors important in rectal cancer: pathologic lymph node involvement and CRM [35]. Because the completeness of tumor resection relies on the assessment of resection margins by the pathologist, knowledge of the CRM before therapy provides important prognostic information for local recurrence and for development of distant metastasis and survival [36].

These three outcomes were registered for 36 patients at the moment of surgery. For all these outcomes, 'responders' are distinguished from 'non-responders'. The grading of regression established by Wheeler and colleagues [34] (from now on referred to as WHEELER) is a modified pathological staging system for irradiated rectal cancer. It includes a measurement of tumor response after preoperative therapy: grade 1, good responsiveness (tumor is sterilized or only microscopic foci of adenocarcinoma remain); grade 2, moderate responsiveness (marked fibrosis but still with a macroscopic tumor); grade 3, poor responsiveness (little or no fibrosis with abundant macroscopic tumor). Tumors are classified as 'responder' when assigned to grade 1 (26 patients) and 'non-responder' when assigned to grade 2 or 3 (10 patients). Response can also be evaluated with the pathologic lymph node stage at surgery (pN-STAGE). The 'responder' class contains 22 patients with no lymph nodes found at surgery while the 'non-responder' class contains 14 patients with at least 1 regional lymph node. CRM was measured according to the guidelines of Quirke *et al. *[37]. CRM was considered positive when the distance between the tumor and the mesorectal fascia was ≤ 2 mm. Tumors with a negative CRM are classified as 'responder' (27 patients), while tumors with a positive CRM belong to the 'non-responder' class (9 patients). Thirteen patients belong to the 'responder' class for all three outcomes, while there is an overlap of two patients between the 'non-responder' classes.

### Data set II: prostate cancer

#### Patients and treatment

We also applied our method to a publicly available data set of prostate cancer. Lapointe and colleagues [30] first profiled gene expression in 71 prostate tumor cases of which 62 were primary and 9 had lymph node metastases. All tumors were removed by radical prostatectomy (that is, the surgical removal of the prostate gland). A cDNA microarray was used, containing 39,711 human cDNAs representing 26,260 mapped genes. Additionally, DNA CNVs were profiled on cDNA microarrays for CGH for 64 prostate tumor cases, among which 55 were primary tumors and 9 had pelvic lymph node metastases. The arrays were obtained from the Stanford Functional Genomics Facility and included 39,632 human cDNAs corresponding to 22,279 genes [31]. Among the primary tumors, the available gene expression and genomics data were in common for 55.

#### Data preprocessing

Median fluorescence ratios were calculated for genes represented by multiple arrayed cDNAs. Missing gene expression values were imputed unsupervised using the k-nearest neighbors method of Troyanskaya *et al. *[38]. The parameter k was set to 15 such that a missing value for a spot S in a sample was estimated as the weighted average of the 15 spots that are most similar to spot S in the remaining samples. The same unsupervised prefiltering as applied on the rectal cancer data set was used for both the microarray and genomics data sets. Features with a variance in the top 50% were retained, reducing the data sets to 6,974 genes and 7,305 CNVs, respectively.

#### Response classification

Two pathological variables, stage and grade, metastasis of the tumor, as well as the outcome after prostatectomy defined as recurrence were considered. For grade (from now on referred to as GRADE), the Gleason Grading system was used, which is based on the most common and second most common architectural patterns of the glands of the tumor [39]. Two groups could be distinguished based on the architecture of the most common pattern: 36 tumors were well differentiated (that is, low-grade), 19 were poorly differentiated (that is, high-grade). According to the extent of the primary tumor (STAGE), 25 samples were of stage T2 (that is, the cancer is confined within one lobe of the prostate gland), while 25 samples were of advanced stage T3 (that is, the tumor has extended through the fibrous tissue surrounding the prostate gland but no other organs are affected). The stage of the remaining five patients was not known. The cancer had metastasized to distant lymph nodes in 12 tumors, while the cancer had not spread beyond the regional lymph nodes in 38 of the tumors (METASTASIS). Tumor recurrence was defined as a rise in prostate-specific antigen of at least 0.07 ng/ml or as occurrence of clinical metastasis (RECURRENCE). Seven tumors recurred while 22 tumors did not. The recurrence status of the remaining 26 patients was not available.

### Kernel methods and weighted least squares support vector machines

Kernel methods are a group of algorithms that can handle a very wide range of data types, such as vectors, sequences, networks, and so on. They map the data *x *from the original input space to a high dimensional feature space with the mapping function Φ(*x*). This embedding into the feature space is performed by a mathematical object *K*(*x*_*k*_, *x*_*l*_), called a 'kernel function'. This function efficiently computes the inner product ⟨Φ(*x*_*k*_), Φ(*x*_*l*_)⟩ between all pairs of data items x_*k *_and x_*l *_in the feature space, resulting in the kernel matrix. The size of this matrix is determined only by the number of data items, whatever the nature or the complexity of these items. For example, a set of 100 patients each characterized by 6,913 gene expression values is still represented by a 100 × 100 kernel matrix [40]. The representation of all data sets by this real-valued square matrix, independent of the nature or complexity of the data to be analyzed, makes kernel methods ideally positioned for heterogeneous data integration.

Any symmetric, positive semidefinite function is a valid kernel function, resulting in many possible kernels - for example, linear, polynomial, and diffusion kernels. They all correspond to a different transformation of the data, meaning that they extract a specific type of information from the data set. In this paper, the normalized linear kernel function:

where  is used instead of the linear kernel function . With the normalized version, the values in the kernel matrix will be bounded because the data points are projected onto the unit sphere while these elements can take very large values without normalization. Normalizing is thus required when combining multiple data sources to guarantee the same order of magnitude for the kernel matrices of the data sets.

A kernel algorithm for supervised classification is the SVM developed by Vapnik [41] and others. Contrary to most other classification methods and due to the way data are represented through kernels, SVMs can tackle high dimensional data (for example microarray data). Given a training set  of N samples with feature vectors *x*_*k *_∈ *R*^*n *^and output labels *y*_*k *_∈ {-1, +1}, the SVM forms a linear discriminant boundary *y*(*x*) = sign[*W*^*T*^Φ(*x*)+*b*] in the feature space with maximum distance between samples of the two considered classes, with *w *representing the weights for the data items in the feature space and *b *the bias term. This corresponds to a non-linear discriminant function in the original input space. A modified version of SVM, LS-SVM, was developed by Suykens *et al. *[3,4]. On high dimensional data sets, this modified version is much faster for classification because a linear system instead of a quadratic programming problem needs to be solved.

The constrained optimization problem for an LS-SVM has the following form:

subject to:

with *e*_*k *_the error variables, tolerating misclassifications in cases of overlapping distributions, and *γ *the regularization parameter, which allows tackling the problem of overfitting. It has been shown that regularization seems to be very important when applying classification methods on high dimensional data [42].

In many two-class problems, data sets are skewed in favor of one class such that the contribution of false negative and false positive errors to the performance assessment criterion are not balanced. We therefore used a weighted LS-SVM in which a different weight *ζ*_*k *_is given to positive and negative samples in order to account for the unbalancedness in the data set [5]. The objective function changes into:

with

and *N*_*P *_and *N*_*N *_representing the number of positive and negative samples, respectively.

### Feature selection

Univariate feature selection techniques are computationally simple but do not incorporate feature-feature interactions. However, due to small sample size limitations, multivariate approaches are often not appropriate for discovering the underlying complex, multivariate correlations. Because it has been shown that univariate gene selection methods lead to good and stable performances across many cancer types and yield in many cases consistently better results than multivariate approaches [43], we used the method DEDS (differential expression via distance synthesis) [44]. This technique is based on the integration of different univariate test statistics via a distance synthesis scheme because features highly ranked simultaneously by multiple statistics are more likely to be differentially expressed than features highly ranked by a single test statistic. The statistical tests combined are ordinary fold changes, ordinary *t*-statistics, SAM (significance analysis for microarrays) statistics and moderated *t*-statistics. DEDS is available as a BioConductor package in R.

We applied DEDS to the microarray data sets as well as the genomics data set. From our experience, DEDS is less appropriate for data with a limited set of features (data not shown). Since the proteomics data on rectal cancer contain only 90-92 cancer-related proteins, one test statistic suffices, for which we chose the Wilcoxon rank sum test.

### Model building

To determine the optimal number of features, we use a leave-one-out (LOO) cross-validation approach in which we increase the number of included features iteratively according to the obtained feature ranking but in which we do not include more features than the number of samples in the data set on which the optimal number of features is determined, as discussed by Li and Yang [45]. Besides the number of features, the parameters of the kernel method (parameter *γ *for LS-SVM with normalized linear kernel) also need to be selected. This selection occurs on a k-dimensional grid with k - 1 the number of data sets included. We considered 40 possible values for *γ*, ranging from 10^-4 ^to 10^6 ^on a logarithmic scale. In each LOO iteration, a sample is left out, feature selection is performed on the remaining n - 1 samples, and models are built for all possible combinations of parameters on this grid. Each model with the instantiated parameters is evaluated on the left out sample. This whole procedure is repeated for all samples. The model parameters are chosen corresponding to the model with the highest LOO area under the receiver operating characteristic (ROC) curve (AUC). If multiple models have the same AUC, the model with the lowest balanced error rate and an as high as possible sum of sensitivity and specificity is chosen. For each considered outcome, the AUC of the best performing model is compared with the AUC of the other models using the method of Hanley and McNeil [46]. The final features are chosen as those that occurred most often in the top rankings determined in each LOO iteration.

**Figure 1:**
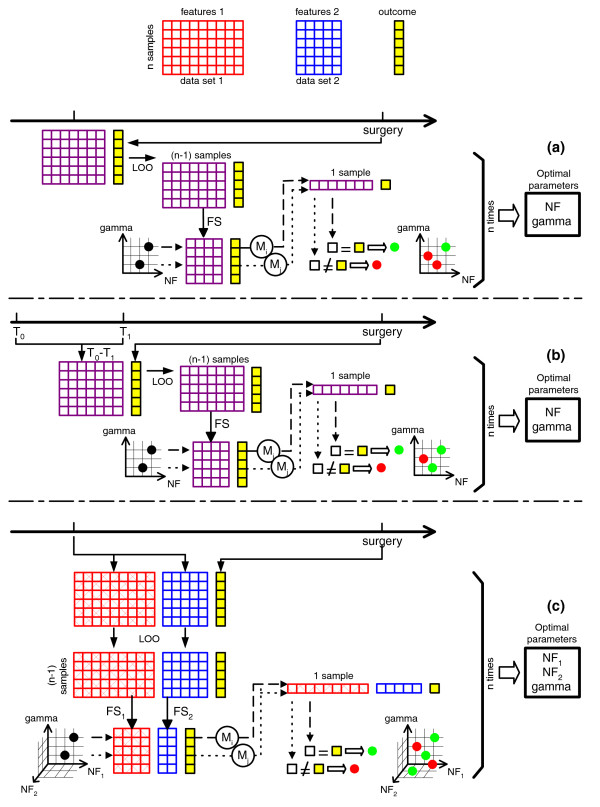
Overview of the three applied model building strategies. **(a) **Use of a single data set; **(b) **manual integration of data over time; **(c) **a genome-wide integration approach. The data sets are represented as matrices with rows corresponding to patients and columns corresponding to genes, proteins, or CNVs. In step A, LS-SVM models are built on each data set separately. A two-dimensional grid is used for the optimization of the regularization parameter and the number of features. For step B, data sets over time are combined. By using the changes in expression or abundance as features, a two-dimensional grid is suficient. In step C, an intermediate integration method is used for the integration of all available data sets. A k-dimensional grid is required for optimizing the regularization parameter and the number of features selected from the (k - 1) integrated data sets. FS, feature selection; M_*i*_, model for parameter combination i; NF, number of features; T, time point.

Three kinds of model building strategies are proposed, different in the degree of integration. Figure 1 shows these strategies in more detail. The data sets are represented as matrices with rows corresponding to patients and columns corresponding to genes, proteins, or CNVs. The matrices representing microarray or genomics data are larger than those for the proteomics data to emphasize the difference in dimensionality.

All three strategies were applied to the microarray and proteomics data sets of rectal cancer. For the prostate cancer data set, however, only two strategies were applicable due to a lack of measurements repeated over time. For all models the parameters were trained according to the same approach, which makes the corresponding LOO results comparable for each outcome separately.

#### Step A models: single data set

In a first step, LS-SVM models are built on each data set separately, mimicking the results that would have been obtained when only static data from one platform were available. For rectal cancer, the single data sets are microarray at *T*_0_, microarray at *T*_1_, proteomics at *T*_0_, and proteomics at *T*_1 _for the prediction of a regression grading system and two prognostic factors (Figure 1a). For prostate cancer, LS-SVM models are built on the microarray and genomics data separately for the prediction of grade, stage, metastasis, and recurrence. Because of only one set of features, a two-dimensional grid is used for the optimization of the regularization parameter and the number of features.

#### Step B models: manual integration of data over time

When measurements are repeated at multiple time points, knowledge over time can be exploited. For rectal cancer, data were available before and early in therapy and, therefore, can be combined in the models. This is done for each data type separately by manually calculating the change in gene expression or protein abundance between the first two time points (*T*_0_-*T*_1_). These changes over time are used as features for the models as shown in Figure 1b. Also for these models, a two-dimensional grid suffices for the optimization of the regularization parameter and the number of features.

#### Step C models: multiple omics integration approach

The previous two types of models (steps A and B) are considered to verify whether complex integration of data over multiple layers of biological regulation is crucial. The ability of kernel methods to deal with complexly structured data makes them ideally positioned for more advanced integration of heterogeneous data sources. We will use the intermediate integration method proposed in [47] in which a kernel matrix is computed for each data source separately. Subsequently, these data sources can be integrated in a straightforward way by summing the multiple kernel matrices. Positive semidefiniteness of the linear combination of kernel matrices is guaranteed by constraining the weights of the kernels to be non-negative. A weighted LS-SVM is trained on the explicitly heterogeneous kernel matrix. The choice of the weights to give to each data set is important. A kernel framework for optimizing weights is proposed in [48]. This optimization is important when dealing with many data sets of which only several are relevant. However, when the number of data sets is limited and most of them are reliable and relevant to the problem at hand, a trade-off needs to be made between performance and computational burden (for example, extra required cross-validation loops). Due to the rather small sample size in both case studies, weights were chosen equally. Moreover, our aim is to emphasize that classification becomes more accurate when data from multiple layers in the genome are available and to offer a machine learning-based method for integrating these data sources, rather than to improve an algorithm for the optimization of weights (for example, [48]). A three-dimensional grid is used for the optimization of the parameters, that is, the regularization parameter, the number of genes selected from the microarray data sets, and the number of proteins or CNVs obtained from the proteomics data sets or the genomics data set, respectively. For the data on rectal cancer, the number of genes/proteins selected at *T*_0 _and *T*_1 _were taken equally when data from both time points were considered. Figure 1c gives an overview of the strategy.

## Results

### Study I: rectal cancer

Using the methodologies shown in Figure 1, models were built using microarray and proteomics data of 36 rectal cancer patients at two time points during therapy for the prediction of three outcomes registered at the moment of surgery: a tumor regression grading system (WHEELER) and two prognostic factors, pathologic N stage at surgery (pN-STAGE) and the circumferential margin involvement (CRM). The models with the highest AUC, lowest balanced error rate and an as high as possible sum of sensitivity and specificity are shown in Table 2. The step A models are *MT*_0 _(model based on microarray data at *T*_0_), *MT*_1 _(model based on microarray data at *T*_1_), *PT*_0 _(model based on proteomics data at *T*_0_), and *PT*_1 _(model based on proteomics data at *T*_1_). The step B models consist of *MT*_0_-*T*_1 _(model based on change in gene expression between *T*_0 _and *T*_1_) and *PT*_0_-*T*_1 _(model based on change in protein abundances between *T*_0 _and *T*_1_). Finally, the step C models comprise *MT*_01 _(model based on microarray data at both time points), *PT*_01 _(model based on proteomics data at both time points), *MPT*_0 _(model based on microarray and proteomics data at *T*_0_), *MPT*_1 _(model based on microarray and proteomics data at *T*_1_), all possible combinations of three data sets (using the same name convention), and *MPT*_01 _(model based on all data (microarray and proteomics data at both time points)). The numbers of genes and proteins were chosen to optimize the LOO performance of the LS-SVM models. The features selected most often in the 36 LOO iterations are listed and discussed. For each outcome, the ROC curve of the best model was compared with the ROC curves of all other models [46]. The *P*-values of these significance tests are reported as well.

**Table 2:** LS-SVM models for the prediction of WHEELER, pN-STAGE and CRM in rectal cancer

Outcome	Model	NG*	NP^†^	AUC (SE)^‡^	*p*-value^§^
**WHEELER**					
A	*MT*_0_	4		0.7538 (0.1085)	0.0987
	*MT*_1_	29		0.9038 (0.0502)	0.6861
	*PT*_0_		35	0.7423 (0.0867)	0.0540
	*PT*_1_		11	0.9038 (0.0575)	0.7273
					
B	*MT*_0_-*T*_1_	32		0.6846 (0.1215)	0.0598
	*PT*_0_-*T*_1_		5	0.8654 (0.0621)	0.4135
					
C	*MT*_01_	3^¶^		0.7808 (0.0985)	0.1320
	*PT*_01_		21^¶^	0.7692 (0.0831)	0.0831
	*MPT*_0_	3	35	0.8461 (0.0718)	0.2760
	*MPT*_1_	**25**	**12**	**0.9269 (0.0425)**	
	*MPT*_01_	2^¶^	31^¶^	0.8846 (0.0558)	0.4858
					
	*MT*_0_*PT*_1_	2	4	0.9385 (0.0444)	0.8101^¥^
					
**pN-STAGE**					
A	*MT*_0_	25		0.6493 (0.0914)	2.315e-4
	*MT*_1_	22		0.8506 (0.0665)	0.0362
	*PT*_0_		2	0.6753 (0.0906)	6.659e-4
	*PT*_1_		12	0.8409 (0.0652)	0.0238
					
B	*MT*_0_-*T*_1_	4		0.6071 (0.0986)	1.359e-4
	*PT*_0_-*T*_1_		9	0.7662 (0.0900)	0.0153
					
C	*MT*_01_	24^¶^		0.9286 (0.0450)	0.1998
	*PT*_01_		34^¶^	0.8182 (0.0695)	0.0145
	*MPT*_0_	27	27	0.9188 (0.0469)	0.1591
	*MPT*_1_	**21**	**14**	**0.9870 (0.0135)**	
	*MPT*_01_	23^¶^	16^¶^	0.9610 (0.0280)	0.3421
					
	*MT*_0_*PT*_01_	26	20^¶^	1 (0)	0.3347^¥^
					
**CRM**					
A	*MT*_0_	33		0.6790 (0.1016)	0.0072
	*MT*_1_	9		0.9259 (0.0472)	0.4955
	*PT*_0_		34	0.8518 (0.0624)	0.0935
	*PT*_1_		34	0.7654 (0.0831)	0.0281
					
B	*MT*_0_-*T*_1_	6		0.9136 (0.0480)	0.4030
	*PT*_0_-*T*_1_		2	0.8272 (0.0709)	0.0849
					
C	*MT*_01_	16^¶^		0.8066 (0.0846)	0.0468
	*PT*_01_		3^¶^	0.7531 (0.0865)	0.0227
	*MPT*_0_	7	27	0.8477 (0.0688)	0.1340
	*MPT*_1_	**7**	**33**	**0.9630 (0.0344)**	
	*MPT*_01_	2^¶^	3^¶^	0.8230 (0.0771)	0.0973
					
	*MT*_1_*PT*_0_	16	14	0.9630 (0.0376)	1
	*MT*_01_*PT*_1_	9^¶^	29	0.9876 (0.0146)	0.4924^¥^

*Number of genes selected in each LOO iteration. ^†^Number of proteins selected in each LOO iteration. ^‡^Area under the ROC curve (standard error) obtained with leave-one-out. ^§^Comparison of AUC between each model and the best model in bold [46]. ^¶^Number of features used at both time points. ^¥^This model is better than the model in bold we compare with.

Table 2 shows the LS-SVM models for the considered combinations of data sets to predict WHEELER, pN-STAGE, and CRM with the optimal number of genes and proteins selected with DEDS and the Wilcoxon rank sum test, respectively. The corresponding ROC curves are shown in Additional data file 1. The performance of the models based on three data sets is given in Additional data file 2. Due to the slightly, but not significantly, better performance for each outcome of one model based on three data sets compared to models based on two data sets, we report the results for the best model combining two data sets. Such models would only require a sample to be taken at one time point (*MPT*_0_, *MPT*_1_) or one technology to be applied on two time points (*MT*_01_, *PT*_01_). For the prediction of WHEELER, the expression of 25 genes and 12 proteins at *T*_1 _was best, although not significantly, with an AUC of 0.9269. Also for pN-STAGE, combining both data sets at *T*_1 _using the expression of 21 genes and 14 proteins resulted in the best LOO AUC of 0.9870. This performance is significantly better than all step A and B models as well as *PT*_01_. Finally, the inclusion of 7 genes and 33 proteins at *T*_1 _led to an AUC of 0.9630 for the prediction of CRM. Four models based on only one data type perform significantly worse compared to *MPT*_1_. For all outcomes, none of the selected proteins are a product of the selected genes.

The contribution of the genes and/or proteins in rectal or colorectal cancer that were selected most often in the LOO iterations of *MPT*_1 _and predicted most accurately WHEELER, pN-STAGE, or CRM are shown in Table 3. A protein important for CRM, for example, is the epidermal growth factor receptor (EGFR), involved in signaling pathways affecting cellular growth, differentiation, and proliferation. This protein represents one of the most promising targets allowing progress in colorectal cancer treatment. It has been suggested that EGFR polymorphisms as well as polymorphisms of other genes active in the EGFR pathway may be potential indicators of radiosensitivity in patients with rectal cancer treated with chemoradiation [49]. In colorectal cancer, pro-inflammatory cytokines such as interleukin-1 beta and interleukin-6 may be accountable for the overexpression of *Cox-2*, important in the early stage and for progression [50]. Transforming growth factor alpha, down-regulated in our patients with a good responsiveness to preoperative therapy, is implicated in metastatic spread of colon cancer cells [51]. The expression of interleukin-8 is associated with induction and progression of colorectal carcinoma and the development of colorectal liver metastases [52]. In our data set, it is down-regulated in the group of patients with no lymph nodes found at surgery. Finally, elevated carcinoembryonic antigen and cancer antigen 19-9 are related to poor outcome in colorectal cancer [53]. Their levels are low in patients with no lymph nodes, while carcinoembryonic antigen is also less expressed in patients with a negative CRM, that is, belonging to the class of 'responders'. A complete list of the genes and proteins chosen by the models *MPT*_1 _are shown, for each outcome separately, in Additional data file 3. The predictions seem to depend on mainly different subsets of features. The gene encoding PAI-2 is important for both WHEELER and CRM, while the proteins important for two of the three outcomes are interleukin-4, ferritin, apolipoprotein H, epidermal growth factor, matrix metalloproteinase-2, and lymphotactin. Notably, these genes and proteins were also selected by the other models based on microarray and/or proteomics data at *T*_1_, although the specific feature ranking depends on the number of features included. Some of these genes and proteins were also included in the models based on data at *T*_0_.

**Table 3:** Features for (colo)rectal cancer selected by *MPT*_1 _and known to be involved in this type of cancer

Outcome*	Gene/protein	Hits^†^	Region	Function	Up/down^‡^	Reference
W	Cox-2	36	1q25.2-q25.3	Progression	Up	[50]
W	IL-1B	36	2q14	Inflammatory response	Up	[50]
W	Ferritin	36	11q13; 19q13.3-q13.4	Iron storage	Down	[63]
W	EGF	36	4q25	Cell growth/proliferation/differentiation	Up	[64]
W	MMP-2	36	16q13-q21	Invasion/metastasis	Up	[65]
W	TGF*α*	36	2p13	Angiogenesis/cell proliferation	Down	[51]
W	SELE	25	1q22-q25	Progression/metastasis	Up	[66]
W	GM-CSF	24	5q31.1	Maintenance of granulocytes/macrophages	Up	[67]
W	MMP-1	15	11q22.3	Tumor invasion/metastasis/poor prognosis	Up	[68]
						
N	Reg4	36	1p13.1-p12	Early carcinogenesis	Down	[69]
N	MUC2	36	11p15.5	Deregulated by TNFα	Down	[70]
N	CA1	36	8q13-q22.1	Carbonate dehydratase activity	Down	[71]
N	CA2	36	8q22	Carbonate dehydratase activity	Down	[71]
N	CLDN8	36	21q22.11	Tumorigenesis	Down	[72]
N	CEA	36	19q13.1-q13.2	Cell adhesion; tumor marker for recurrence	Down	[53]
N	IL-1ra	36	2q14.2	Carcinogenesis	Up	[73]
N	CA19-9	36		Tumor marker for recurrence	Down	[53]
N	Ferritin	36	11q13; 19q13.3-q13.4	Iron storage	Down	[63]
N	IL-1beta	36	2q14	Inflammatory response	Down	[50]
N	beta2-microglobulin	36	15q21-q22.2	Metastasis	Up	[74]
N	RARRES1	31	3q25.32-q25.33	Cell proliferation	Down	[75]
N	IL-8	28	4q13-q21	Progression/metastasis	Down	[52]
N	TNFRII	24	1p36.3-p36.2	Apoptosis	Up	[76]
						
C	ICAM-1	36	19p13.3-p13.2	Metastasis	Down	[77]
C	CEA	36	19q13.1-q13.2	Cell adhesion; tumor marker for recurrence	Down	[53]
C	MMP-2	36	16q13-q21	Invasion/metastasis	Up	[65]
C	Adiponectin	36	3q27	Metabolic/hormonal processes	Down	[78]
C	Thrombospondin-1	36	15q15	Angiogenesis/tumor growth	Up	[79]
C	EGFR	36	7p12	Cell growth/proliferation/differentiation	Up	[49]
C	Tissue factor	35	1p22-p21	Angiogenesis/metastasis	Up	[80]
C	CYP1B1	35	2p21	Drug metabolism	Down	[81]
C	EGF	32	4q25	Cell growth/proliferation/differentiation	Up	[64]

*W, WHEELER; N, pN-STAGE; C, CRM. ^†^Number of occurrences of the gene/protein in the 36 LOO iterations. ^‡^Up/down-regulation in the good responders with respect to moderate or poor responders; no lymph nodes with respect to at least one regional lymph node; negative CRM with respect to positive CRM. CRC, (colo)rectal cancer.

### Study II: prostate cancer

The same methodology was applied to microarray and genomics data of 55 patients with prostate cancer. Table 4 shows the results for the prediction of the grade and stage of the tumor (GRADE and STAGE), as well as the tumors that metastasized to distant lymph nodes (METASTASIS) or that recurred (RECURRENCE). Because the data were gathered at one time point, only step A and C models are applicable. The step A models are represented as *M *(model based on microarray data) and *G *(model based on genomics data), and the step C model based on both microarray and genomics data as *MG*. Also, after having optimized the essential number of features to be included using a LOO cross-validation, the final genes and CNVs were selected based on their position and number of occurrences in the 55 LOO rankings.

**Table 4:** LS-SVM models for the prediction of GRADE, STAGE, METASTASIS and RECURRENCE in prostate cancer

Outcome	Model	NG*	NC^†^	AUC (SE)^‡^	*p*-value^§^
**GRADE**					
A	*M*	24		0.8304 (0.0623)	0.2727
	*G*		8	0.7822 (0.0632)	0.0503
C	*MG*	**6**	**8**	**0.9006 (0.0413)**	
					
**STAGE**					
A	*M*	18		0.6576 (0.0778)	0.0191
	*G*		32	0.7936 (0.0631)	0.3466
C	*MG*	**42**	**22**	**0.8528 (0.0550)**	
					
**METASTASIS**					
A	*M*	18		0.9759 (0.0178)	0.4392
	*G*		12	0.8114 (0.0755)	0.0166
C	*MG*	**18**	**3**	**0.9868 (0.0121)**	
					
**RECURRENCE**					
A	*M*	24		0.7208 (0.0936)	0.5392
	*G*		26	0.4481 (0.1433)	0.0354
C	*MG*	**32**	**2**	**0.7857 (0.0934)**	

*Number of genes selected in each LOO iteration. ^†^Number of copy number variations selected in each LOO iteration. ^‡^Area under the ROC curve (standard error) obtained with leave-one-out. ^§^Comparison of AUC between each model and the best model in bold [46].

We obtained similar results as for rectal cancer. Combining gene expression with measurements at the DNA level (*MG*) led, for all four outcomes, to an improvement in classification accuracy and was significant in some cases (Table 4). For the prediction of GRADE, six genes and eight CNVs selected with DEDS resulted in an AUC of 0.9006. For STAGE, 42 genes and 22 CNVs were needed for a performance of 0.8528. The model *MG *for the prediction of METASTASIS had an AUC of 0.9868 when fusing the expression of 18 genes with 3 CNVs. Finally, the prediction of RECURRENCE was most difficult, with an AUC of 0.7857 when combining 32 genes and 2 CNVs. Additional data file 1 shows the ROC curves of the models listed in Table 4.

Several genes and CNVs have been selected by *MG *and are known to be involved in, and important for, prostate cancer (Table 5). The gene *ALOX15B *is a suppressor of prostate tumor development [54] and in this data set is down-regulated in tumors of high-grade and in tumors that recurred. Both *SFRP4 *and *CXCL14 *on the other hand are inhibitors of prostate tumor growth [55,56]. *SFRP4 *is up-regulated in tumors of high-grade, and *CXCL14 *in tumors of advanced stage. A small deletion involving chromosomal band 21q22.3 fuses all coding exons of *ERG *to androgen-related sequences in the promoter of the prostate-specific *TMPRSS2 *gene. This chromosomal rearrangement is a highly prevalent oncogenic alteration in prostate tumor cells and leads to an aberrant expression of the *ERG *proto-oncogene, important for early prostate carcinogenesis [57]. In this data set, *ERG *is overexpressed in tumors in which the cancer metastasized to distant lymph nodes. It has been shown that this genetic biomarker is a strong prognostic factor for disease recurrence, and can be used for early detection and outcome prediction in prostate cancer [58]. *VAV3*, an oncogene involved in development and progression of prostate cancer, is up-regulated in tumors that metastasized [59]. It has previously been shown that strong overexpression of *TIAM1 *is significantly associated with disease recurrence and a decreased disease-free survival [60]. Also, *JAG1 *is significantly associated with recurrence [61] and plays a role in cell growth, progression, and metastasis. In this data set, both genes are up-regulated in the group of tumors that recurred. Finally, several germline mutations or variants in *RNASEL *have been observed among hereditary prostate cancer cases, indicating that polymorphic changes within the *RNASEL *gene may be associated with increased risk of familial but not sporadic prostate cancer [62]. A list of all the genes and CNVs selected by the models *MG *are shown in Additional data file 3. As for rectal cancer, the outcomes for prostate cancer seem to be characterized by mainly different sets of features. Five genes overlap between at least two outcomes (*ERG*, *AHSG*, *SEMA4G*, *F5*, and *ALOX15B*), while the same holds for four CNVs of the genes *GPD1L*, *KCTD12*, *SMYD5*, and *TRO*.

**Table 5:** Features for prostate cancer selected by *MG *and known to be involved in this type of cancer

Outcome*	Gene/CNV	Hits^†^	Region	Function	Up/down^‡^	Reference
G	*SFRP4*	55	7p14.1	Inhibitor of PT growth/invasion	Up	[55]
G	*VCAN*	55	5q14.3	Contributor to PC pathology	Up	[82]
G	*ALOX15B*	36	17p13.1	Suppressor of PT development	Down	[54]
						
S	*MAGEA4*	50	Xq28	Only expressed in PC (diagnosis and therapy)	Down	[83]
S	*ANPEP*	50	15q25-q26	PT cell invasion	Down	[84]
S	*POU4F1*	50	13q31.1	PC cell growth	Down	[85]
S	*CXCL14*	48	5q31	Inhibitor of PT growth	Up	[56]
S	*RNASEL*	48	1q25	Polymorphic changes as tumor; suppressor in hereditary PC	Up	[62]
S	*GDEP*	41	4q21.1	Prostate-specific gene	Down	[86]
						
M	*ERG*	50	21q22.3	Proto-oncogene; early prostate carcinogenesis	Up	[57]
M	*AREG*	49	4q13-q21	PC progression/growth via TARP	Down	[87]
M	*VAV3*	49	1p13.3	Oncogene; PC development/progression	Up	[59]
M	*ADAMTS1*	26	21q21.2	Negatively affected by TGFbeta1, which increases VCAN-expression	Down	[82]
						
R	*AZGP1*	29	7q22.1	Inversely associated to tumor stage; predictor of biochemical recurrence	Down	[88]
R	*TIAM1*	29	21q22.1-11	Predictor of decreased disease-free survival/recurrence	Up	[60]
R	*FGG*	28	4q28	PC cell growth	Down	[89]
R	*ATF3*	26	1q32.3	Inversely related to invasion/angiogenesis; positively correlated to metastases	Down	[90]
R	*JAG1*	26	20p12.1-11.23	Cell growth/progression/metastasis	Up	[61]
R	*ERG*	14	21q22.3	Proto-oncogene; early prostate carcinogenesis	Up	[57]
R	*ALOX15B*	14	17p13.1	Suppressor of PT development	Down	[54]

*G, GRADE; S, STAGE; M, METASTASIS; R, RECURRENCE. ^†^Number of occurrences of the gene/CNV in all LOO iterations (number of LOO iterations for G = 55, S = 50, M = 50, R = 29). ^‡^Up/down-regulation in high-grade with respect to low-grade; advanced stage with respect to early stage; metastasis with respect to no metastasis; recurrence with respect to no recurrence. PC, prostate cancer; PT, prostate tumor.

### Comparison with an ensemble approach

To assess the benefit of our kernel-based integration approach over standard data fusion techniques, we implemented an ensemble approach in which each data set gives rise to a separate LS-SVM classifier. These individual LS-SVM models were built similarly to the step A models, with the same number of genes, proteins or CNVs selected as included in the best models *MPT*_1 _and *MG*. Subsequently, as a late integration step, the continuous outputs of these models were added.

For the study on rectal cancer, the AUC values of the ensemble models integrating the microarray and proteomics data set gathered at *T*_1_, and the corresponding AUC values of the best model obtained with our strategy (*MPT*_1_) are shown in Table 6. The *P*-values of the significance tests comparing the ROC curves are reported as well [46]. For CRM, our strategy was significantly better than the ensemble approach at a significance level of 0.05. For WHEELER and pN-STAGE, the AUC values did not differ significantly. Similarly for the study on prostate cancer, the AUC values of *MG *were compared with the AUC values of the ensemble models combining microarray and genomics data (Table 6). For all four outcomes, the AUC of *MG *was better than the AUC of the ensemble models, although being significantly better for RECURRENCE only.

**Table 6:** Comparison of our kernel-based integration approach with the ensemble approach

Outcome	AUC (SE)*:*MPT*_1_/*MG*	AUC (SE)*: ensemble approach	*p*-value
WHEELER	0.9269 (0.0425)	0.9500 (0.0339)	0.6160
pN-STAGE	0.9870 (0.0135)	0.9253 (0.0432)	0.1422
CRM	0.9630 (0.0344)	0.7860 (0.0783)	**0.0384**
			
GRADE	0.9006 (0.0413)	0.8567 (0.0521)	0.3745
STAGE	0.8528 (0.0550)	0.8304 (0.0582)	0.6836
METASTASIS	0.9868 (0.0121)	0.9452 (0.0309)	0.1313
RECURRENCE	0.7857 (0.0934)	0.4545 (0.1352)	**0.0182**

*Area under the ROC curve (standard error) obtained with leave-one-out. ^†^Comparison in AUC between the best models obtained with our strategy (*MPT*_1 _for rectal cancer, *MG *for prostate cancer) and the corresponding ensemble models based on the same number of features [46]

### Correlation analysis

We additionally verified whether, in both cases, data from multiple layers of molecular biology were complementary. After mapping the entities of the data sets based on their entrez gene IDs, we investigated the correlation between the microarray and proteomics data of rectal cancer on the one hand, and between the microarray and genomics data of prostate cancer on the other hand. Using the Spearman correlation coefficient, there was no significant correlation for rectal cancer between the abundances of the 90-92 proteins and their corresponding transcripts at a significance level of 0.05. The microarray and genomics data sets for prostate cancer were slightly more correlated. While for GRADE the 6 genes selected by the model *MG *did not correlate with their DNA expression, 2 of the 42 selected genes for STAGE were significantly correlated (*P *< 0.05). For METASTASIS and RECURRENCE, there was a significant correlation for one and three genes, respectively. The regions, with involved CNVs selected from the genomics data, were also compared with the regions in which the selected genes from the microarray data were located. For the majority of regions, there was no overlap. For the other regions with the same rough chromosomal location, the genes selected by both data sets were different.

## Discussion

The proposed integration approach has been applied to two patient data sets, each with two high-throughput data sources. Microarray and proteomics were gathered from 36 patients with rectal cancer at two time points during preoperative treatment, while microarray and genomics were gathered from 55 patients with prostate cancer. To verify the merit of our integration approach over the use of a single omics data source, models were built for classifying cancer patients according to therapy response, prognostic factors, metastasis, or recurrence. In many studies, only single data sources are explored for the development of such profiles. However, in our opinion, a single layer of molecular information is inadequate to explain the complete network of molecules underlying a disease. In this study, LS-SVMs were first built on all data sets individually (Figure 1). Next, we manually integrated data measured at multiple time points by building LS-SVMs using the change in expression between two time points. Because the integration of data may be more complex than the change in expression over time, we subsequently applied an intermediate integration approach in which data from multiple omics were combined at the kernel level within the patient domain.

For the data on rectal cancer, all three outcomes - a tumor regression grading system and two prognostic factors - could be predicted most accurately and most cost-efficiently with an AUC ranging from 0.9269 to 0.9870 when fusing microarray and proteomics data gathered during therapy (*MPT*_1_; Table 2). For WHEELER, for example, *MPT*_0 _performance is better than each of the models based on data from an individual technology (*MT*_0 _and *PT*_0_), as is the case for *MPT*_01 _compared to *MT*_01 _and *PT*_01_. This trend of increased performance when combining data from two different technologies was further confirmed by our second data set for prostate cancer patients. Best results for the prediction of grade, stage, metastasis, and recurrence were obtained when integrating microarray and genomics data (*MG*). The corresponding AUC values were 0.9006, 0.8528, 0.9868, and 0.7857, respectively (Table 4). For many of the genes, proteins, and CNVs included in these models, involvement in rectal or prostate cancer has been defined, indicating the reliability of the selected features (Tables 3 and 5). These models were compared with models obtained with an ensemble approach in which classiffiers are combined instead of data sets at the kernel level. Globally, our approach performed better, although not always significantly (Table 6).

By looking at the correlation between two data sets gathered from the same set of patients, we show that data from different layers are mainly complementary. For rectal cancer, there was a lack of correlation between the selected genes and their corresponding proteins. Also, the selected proteins did not significantly correlate with their transcript level, suggesting alternative splicing and post-translational modification. With newer technologies such as mass spectrometry, the whole proteome will become measurable. For prostate cancer, up to three genes included in the model *MG *were significantly correlated with their corresponding CNV.

More specific for the study on rectal cancer, we can conclude from Table 2 that data gathered after an initial dose of cetuximab are more informative for prediction of therapy response than data gathered before the start of the therapy. Neither microarray nor proteomics data can predict the outcomes more accurately at *T*_0 _than *T*_1_, except for the proteomics data at *T*_0 _being more informative for the prediction of CRM. Moreover, when combining both data types at one time point (*MPT*_0 _and *MPT*_1_), the models applicable after the initial dose of cetuximab outperform those at *T*_0_.

We acknowledge that the models proposed in this manuscript are quite expensive. Applying a model for rectal cancer would require microarray and/or proteomics data, gathered at one or two time points during therapy. However, we have attempted to keep the cost to a minimum. The performance difference between models combining two data sets, only requiring a sample to be taken at one time point or one technology to be applied at two time points, and models requiring a sample to be taken at both time points and both technologies to be performed was minimal and not statistically significant. We therefore chose the best model among the models based on two data sets. We admit that there may exist other, less expensive data sources that can contain complementary information as well. Firstly, clinical information is routinely gathered during therapy, such as tumor size, tumor location and number of positive lymph nodes. However, we only had access to the clinical parameter age, for which we performed an additional analysis to verify whether this parameter could be of use. A univariate analysis based on the Wilcoxon rank sum test showed no significant difference in age between the two classes of samples according to the considered outcomes. In a multivariate logistic regression model, the parameter age was not significant as well. Secondly, there is an increasing need for multi-modal studies in which, among others, clinical, genomic and genetic data are collected. Also, imaging, such as computed tomography (CT) and magnetic resonance imaging (MRI) can be a potential predictor to use in combination with high-throughput data sources. Such studies are required to determine which data sets are most relevant for the problem at hand and which data sets should be combined to become good performing, affordable models that are clinically applicable.

## Conclusions

The results suggest that the use of our integration approach on experimental data from multiple levels in the genome can improve the performance of decision support in cancer. For both data sets studied in this manuscript, combining high-throughput data sets (transcriptomics with proteomics, or genomics with transcriptomics) outperformed the models based on data from a single layer of biological information, independent of the outcome considered for prediction. These results emphasize the need for comprehensive multi-modal data gathered with high-throughput technologies as well as imaging, because it is unknown which technologies, and thus which levels of molecular biology, are the most relevant for prognostic prediction. We acknowledge that this will substantially increase costs in a first exploratory phase. However, this is a necessary investment to ultimately obtain cost-efficient models usable in patient tailored therapy.

In the near future, we will compare our kernel-based integration method with a Bayesian network integration framework. These frameworks are complementary. We also plan to apply an ensemble approach for integrating these two frameworks because more accurate classifiers are not only obtained by combining different data types but also by combining individual decisions of multiple classifiers. In this way, the advantages of both methods can be exploited.

## Abbreviations

AUC: area under the ROC curve; CGH: comparative genomic hybridization; CNV: copy number variation; CRM: circumferential margin involvement; DEDS: differential expression via distance synthesis; EGFR: epidermal growth factor receptor; *G*: model based on genomics data; LOO: leave-one-out; LS-SVM: least squares support vector machine; *M*: model based on microarray data; *MG*: model based on both microarray and genomics data; *MPT*_0_: model based on microarray and proteomics data at *T*_0_; *MPT*_1_: model based on microarray and proteomics data at *T*_1_; *MPT*_01_: model based on all data (microarray and proteomics data at both timepoints); *MT*_0_: model based on microarray data at *T*_0_; *MT*_1_: model based on microarray data at *T*_1_; *MT*_01_: model based on microarray data at both time points; *MT*_0_-*T*_1_: model based on change in gene expression between *T*_0 _and *T*_1_; *PT*_0_: model based on proteomics data at *T*_0_; *PT*_1_: model based on proteomics data at *T*_1_; *PT*_01_: model based on proteomics data at both time points; *PT*_0_-*T*_1_: model based on change in protein abundances between *T*_0 _and *T*_1_; ROC: receiver operating characteristic; SVM: support vector machine;*T*_0_: time point before treatment; *T*_1_: time point after the first loading dose of cetuximab but before the start of radiotherapy with capecitabine; *T*_2_: time point at moment of surgery.

## Competing interests

The authors declare that they have no competing interests.

## Authors' contributions

ADa performed the kernel-based integration modeling and drafted the manuscript. OG, FO, and JS participated in the design and implementation of the framework. ADa and OG performed pre-processing of the data. OG, JS, and BDM helped draft the manuscript. ADe, JPM, and KH provided clinical input, looked up patient records in the database, performed sample annotation, and gathered follow-up of patients. All authors read and approved the final manuscript.

## Additional data files

The following additional data files are available with the online version of this paper. Additional data file 1 shows the ROC curves of the optimal LS-SVM models for all considered combinations of data sets shown in Tables 2 and 4. Additional data file 2 shows the results for the prediction of WHEELER, pN-STAGE, and CRM in rectal cancer, using step C models for which a sample is required at both time points and for which both technologies need to be performed. Additional data file 3 contains additional tables 1-3 showing all genes and proteins selected by the best performing models MPT1 for the prediction of WHEELER, pN-STAGE, and CRM in rectal cancer. Additional data file 3 also contains additional tables 4-7 showing, for prostate cancer, the genes and CNVs selected by the best performing models MG for the prediction of GRADE, STAGE, METASTASIS, and RECURRENCE. All tables in additional data file 3 show the number of LOO iterations in which each gene, protein, or CNV was selected, their chromosomal region, and whether it is up- or down-regulated.

## Supplementary Material

Additional data file 1 The ROC curves of the optimal LS-SVM models for all considered combinations of data sets shown in Tables 2 and 4 are shown. Additional Figures 1-3 show the ROC curves for the prediction of WHEELER, pN-STAGE, and CRM in rectal cancer, respectively. For prostate cancer, the ROC curves for the prediction of GRADE, STAGE, METASTASIS, and RECURRENCE are shown in additional Figures 4-7, respectively. Click here for file

Additional data file 2 The results for the prediction of WHEELER, pN-STAGE, and CRM in rectal cancer, using step C models for which a sample is required at both time points and for which both technologies need to be performed. The AUC value and the number of included features are shown for each model. Significance tests were performed to compare these models with the best model based on two data sets shown in bold in Table 2. Click here for file

Additional data file 3 Additional Tables 1-3 show all genes and proteins selected by the best performing models *MPT*_1 _for the prediction of WHEELER (25 genes, 12 proteins), pN-STAGE (21 genes, 14 proteins), and CRM (7 genes, 33 proteins) in rectal cancer. Additional Tables 4-7 show, for prostate cancer, the genes and CNVs selected by the best performing models *MG *for the prediction of GRADE (6 genes, 8 CNVs), STAGE (42 genes, 22 CNVs), METASTASIS (18 genes, 3 CNVs), and RECURRENCE (32 genes, 2 CNVs). All tables additionally show the number of LOO iterations in which each gene, protein, or CNV was selected, their chromosomal region, and whether it is up- or down-regulated. Click here for file
