# Supplementary material for: A kernel-based integration of genome-wide data for clinical decision support
Source: Genome Med. 2009 Apr 3;1(4):39. doi: 10.1186/gm39 (PMC2684660; doi:10.1186/gm39)
Supplement: Additional data file 1 — The ROC curves of the optimal LS-SVM models for all considered combinations of data sets shown in Tables 2 and 4 are shown. Additional Figures 1-3 show the ROC curves for the prediction of WHEELER, pN-STAGE, and CRM in rectal cancer, respectively. For prostate cancer, the ROC curves for the prediction of GRADE, STAGE, METASTASIS, and RECURRENCE are shown in additional Figures 4-7, respectively. [file gm39-S1.pdf]

Additional file 1 - Comparison of the leave-one-out and test performances

The additional figures show the internal leave-one-out (LOO) performances (AUC) corresponding to the optimal number of selected features, ranging from 1 to 35 in steps of 5, for all LS-SVM models based on different combinations of data sources. These boxplots are compared with their respectively test performances. Additional figures 1 to 3 show the comparisons for data set I on rectal cancer, while the results for data set II on prostate cancer are represented in additional figures 4 to 7.

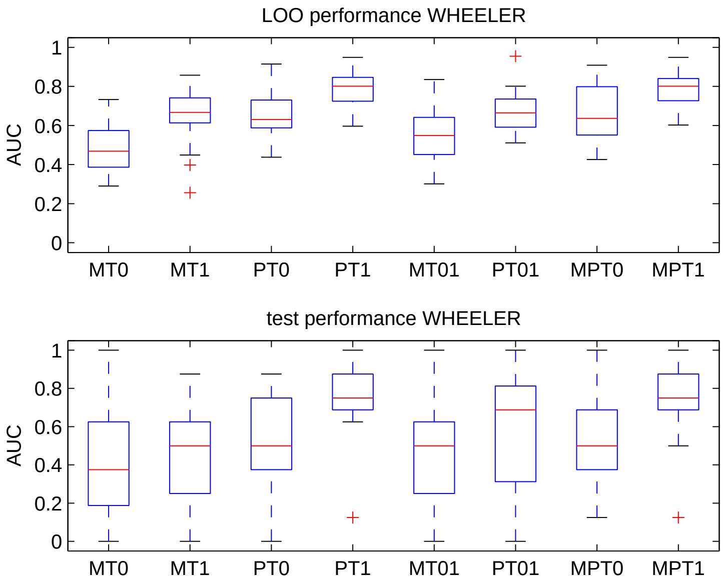

Figure 1: WHEELER – comparison of the internal LOO and test performances of MT_0 (30 genes), MT_1 (25 genes), PT_0 (30 proteins), PT_1 (10 proteins), MT_{01} (5 genes), PT_{01} (15 proteins), MPT_0 (5 genes, 35 proteins), and MPT_1 (1 gene, 10 proteins)

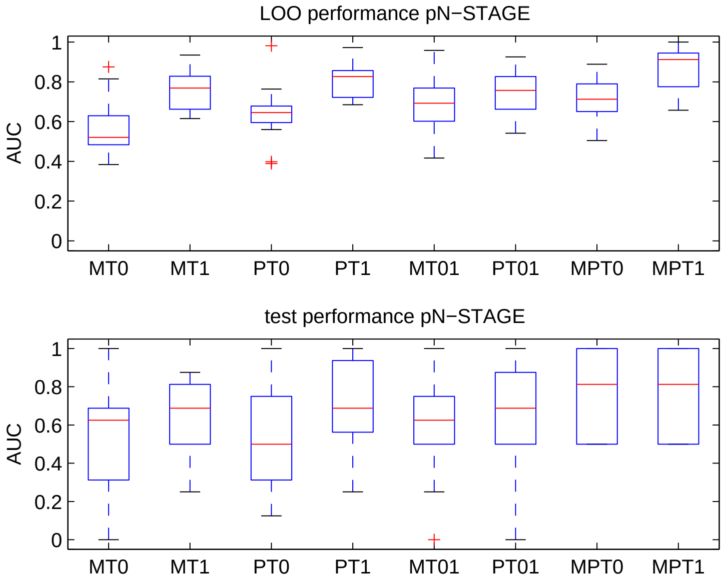

Figure 2: pN-STAGE – comparison of the internal LOO and test performances of MT_0 (30 genes), MT_1 (35 genes), PT_0 (30 proteins), PT_1 (25 proteins), MT_{01} (35 genes), PT_{01} (25 proteins), MPT_0 (25 genes, 25 proteins), and MPT_1 (30 genes, 20 proteins)

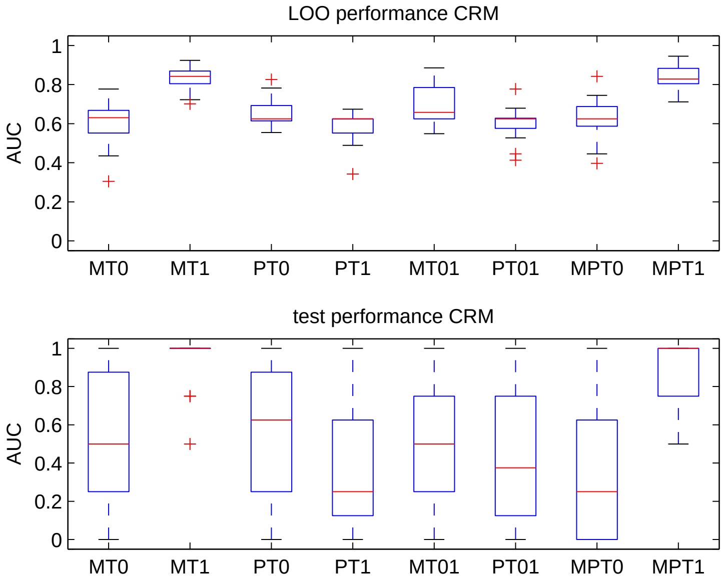

Figure 3: CRM – comparison of the internal LOO and test performances of MT_0 (30 genes), MT_1 (25 genes), PT_0 (30 proteins), PT_1 (30 proteins), MT_{01} (15 genes), PT_{01} (15 proteins), MPT_0 (20 genes, 10 proteins), and MPT_1 (30 genes, 1 protein)

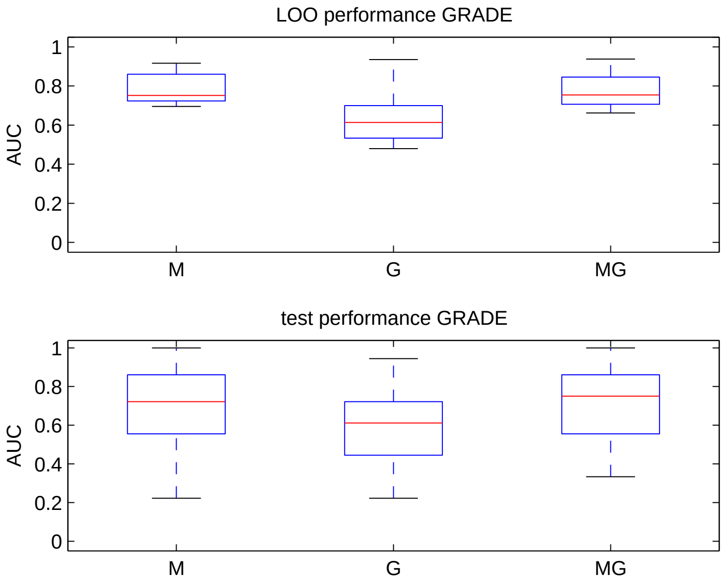

Figure 4: GRADE – comparison of the internal LOO and test performances of M (30 genes), G (20 CNVs), and MG (35 genes, 1 CNV)

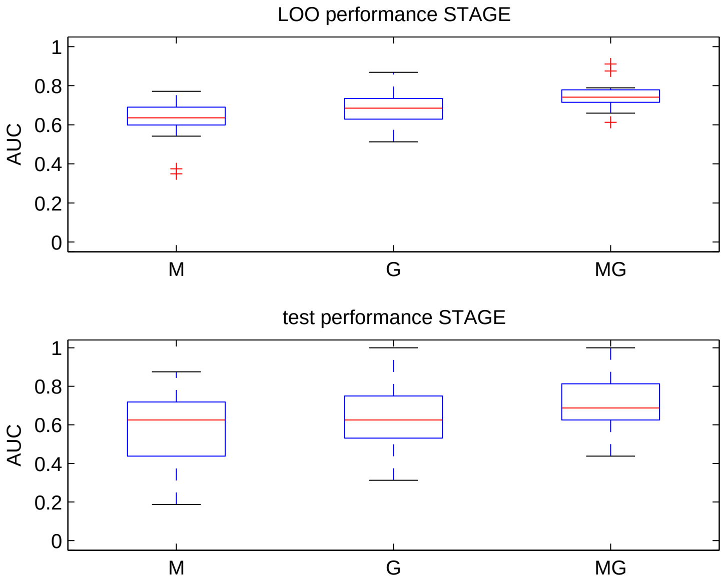

Figure 5: STAGE – comparison of the internal LOO and test performances of M (35 genes), G (35 CNVs), and MG (30 genes, 35 CNVs)

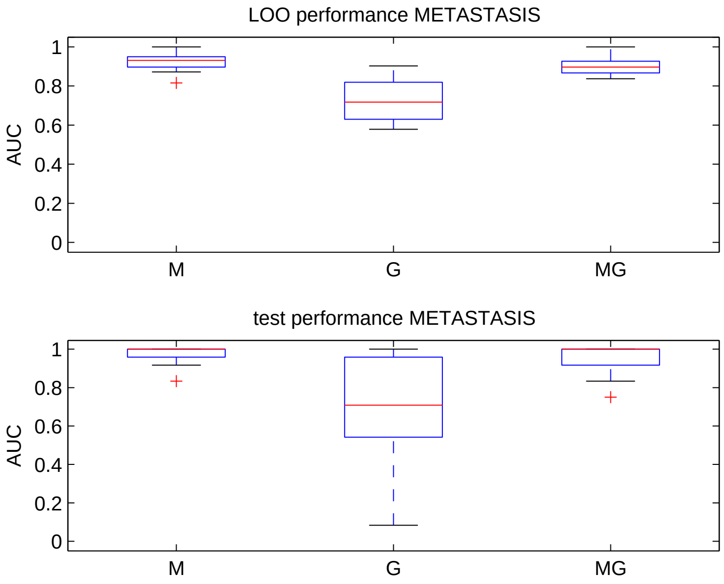

Figure 6: METASTASIS – comparison of the internal LOO and test performances of M (20 genes), G (35 CNVs), and MG (25 genes, 1 CNV)

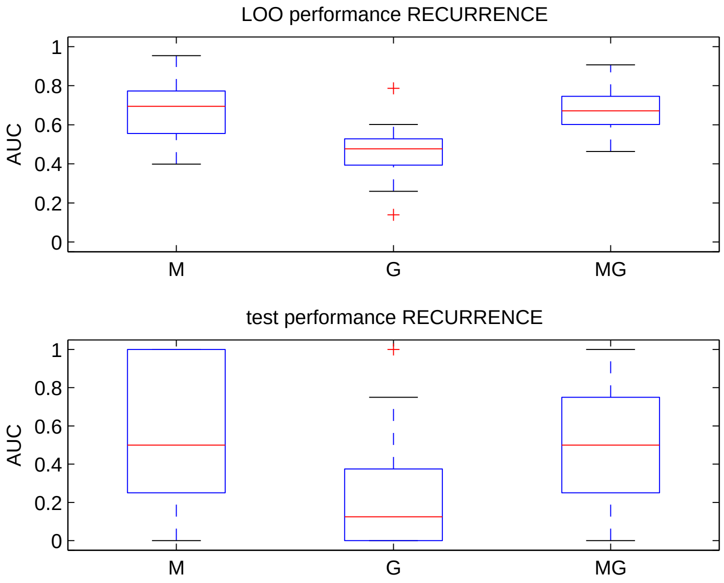

Figure 7: RECURRENCE – comparison of the internal LOO and test performances of M (25 genes), G (20 CNVs), and MG (25 genes, 1 CNV)
