# Supplementary material for: A kernel-based integration of genome-wide data for clinical decision support
Source: Genome Med. 2009 Apr 3;1(4):39. doi: 10.1186/gm39 (PMC2684660; doi:10.1186/gm39)
Supplement: Additional data file 2 — The results for the prediction of WHEELER, pN-STAGE, and CRM in rectal cancer, using step C models for which a sample is required at both time points and for which both technologies need to be performed. The AUC value and the number of included features are shown for each model. Significance tests were performed to compare these models with the best model based on two data sets shown in bold in Table 2. [file gm39-S2.pdf]

Additional file 2 - ROC curves of the models shown in Tables 2 and 4

The ROC curves of the optimal LS-SVM models for all considered combinations of data sets shown in Tables 2 and 4 are shown in these additional figures. Additional figures 1 to 3 show the ROC curves for the prediction of WHEELER, pN-STAGE, and CRM in rectal cancer, respectively. For prostate cancer, the ROC curves for the prediction of GRADE, STAGE, METASTASIS, and RECURRENCE are shown in additional figures 4 to 7, respectively.

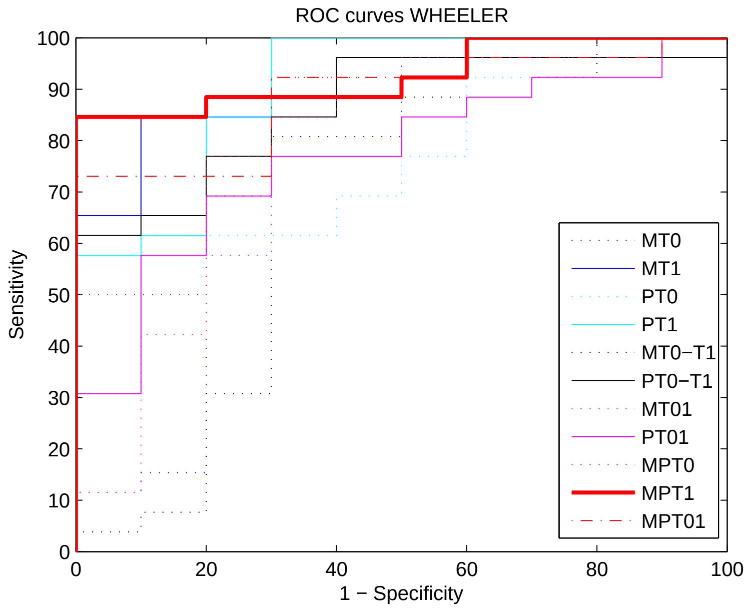

Figure 1: WHEELER – MT_0 (4 genes, blue dotted), MT_1 (29 genes, blue solid), PT_0 (35 proteins, cyan dotted), PT_1 (11 proteins, cyan solid), $MT_0 - T_1$ (32 genes, black dotted), $PT_0 - T_1$ (5 proteins, black solid), MT_{01} (3 genes, magenta dotted), PT_{01} (21 proteins, magenta solid), MPT_0 (3 genes, 35 proteins, red dotted), MPT_1 (25 genes, 12 proteins, red solid), and MPT_{01} (2 genes, 31 proteins, red dashdotted)

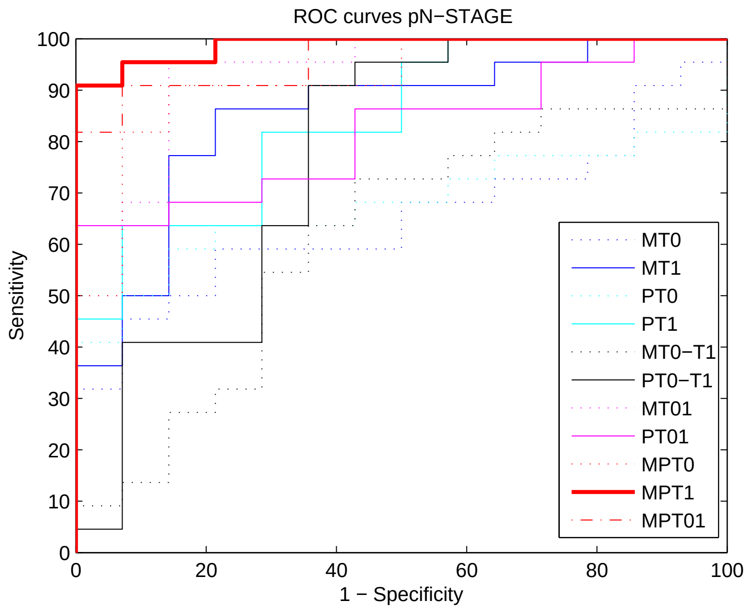

Figure 2: pN-STAGE – MT_0 (25 genes, blue dotted), MT_1 (22 genes, blue solid), PT_0 (2 proteins, cyan dotted), PT_1 (12 proteins, cyan solid), $MT_0 - T_1$ (4 genes, black dotted), $PT_0 - T_1$ (9 proteins, black solid), MT_{01} (24 genes, magenta dotted), PT_{01} (34 proteins, magenta solid), MPT_0 (27 genes, 27 proteins, red dotted), MPT_1 (21 genes, 14 proteins, red solid), and MPT_{01} (23 genes, 16 proteins, red dashdotted)

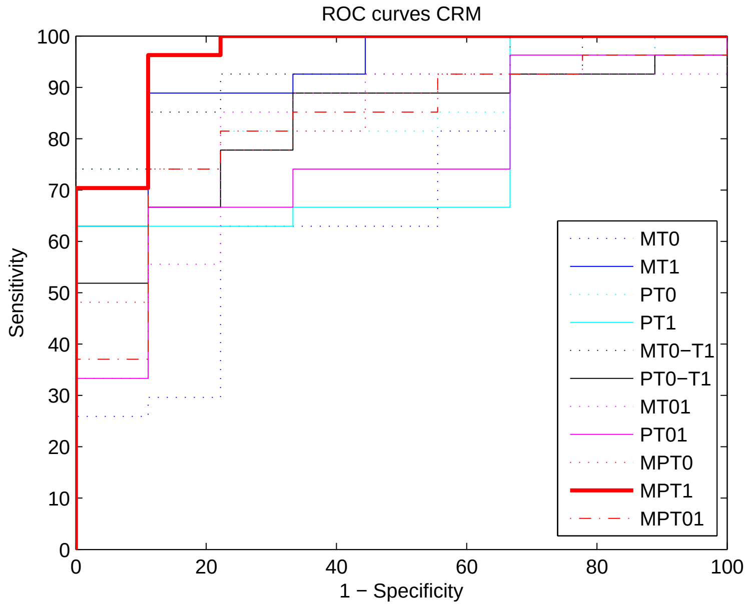

Figure 3: CRM – MT_0 (33 genes, blue dotted), MT_1 (9 genes, blue solid), PT_0 (34 proteins, cyan dotted), PT_1 (34 proteins, cyan solid), $MT_0 - T_1$ (6 genes, black dotted), $PT_0 - T_1$ (2 proteins, black solid), MT_{01} (16 genes, magenta dotted), PT_{01} (3 proteins, magenta solid), MPT_0 (7 genes, 27 proteins, red dotted), MPT_1 (7 genes, 33 proteins, red solid), and MPT_{01} (2 genes, 3 proteins, red dashdotted)

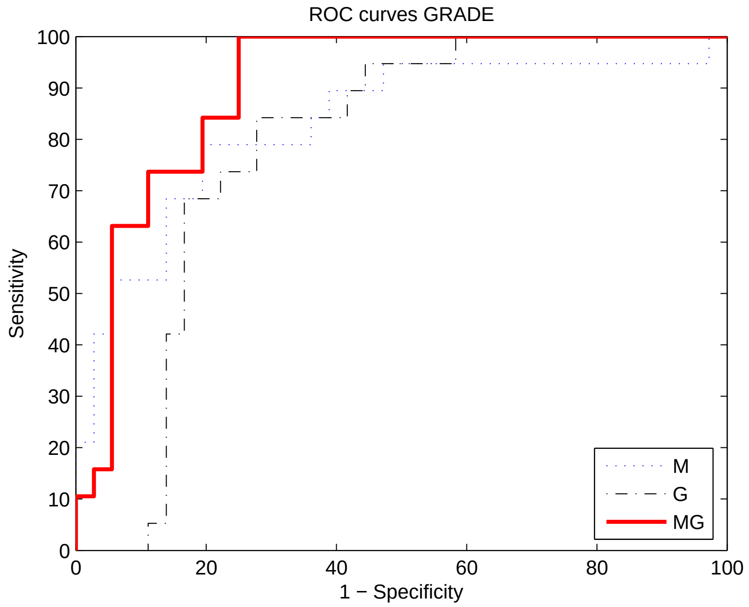

Figure 4: GRADE – M (24 genes, blue dotted), G (8 CNVs, black dashdotted), and MG (6 genes, 8 CNV, red solid)

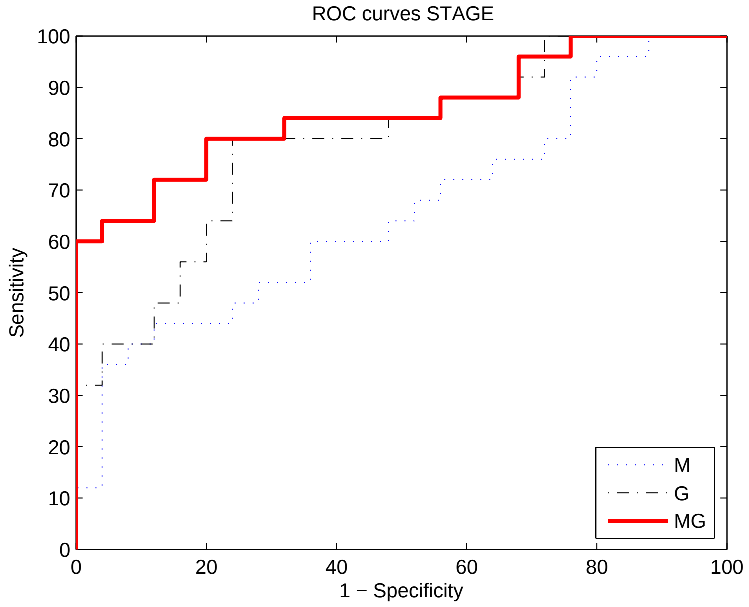

Figure 5: STAGE – M (18 genes, blue dotted), G (32 CNVs, black dashdotted), and MG (42 genes, 22 CNV, red solid)

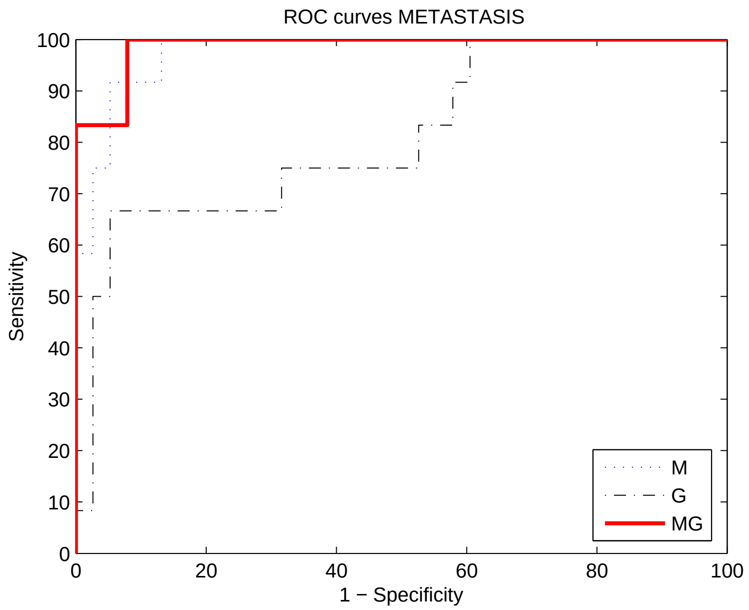

Figure 6: METASTASIS – M (18 genes, blue dotted), G (12 CNVs, black dashdotted), and MG (18 genes, 3 CNV, red solid)

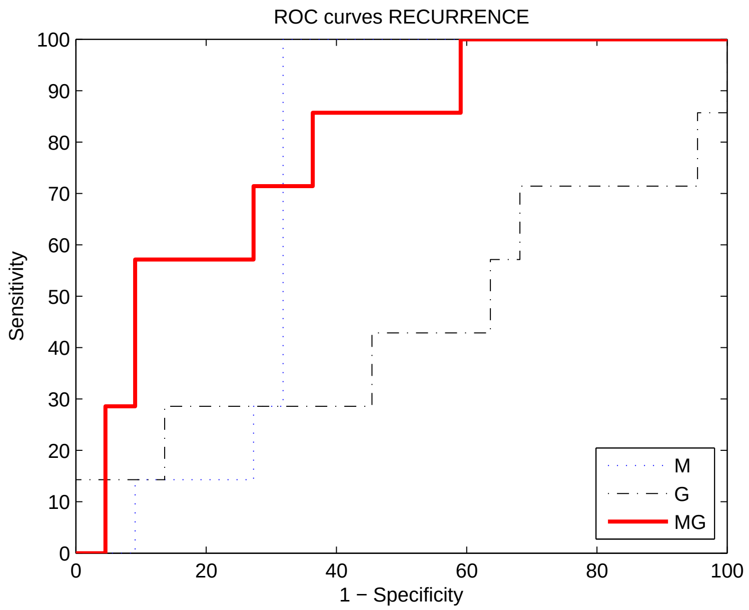

Figure 7: RECURRENCE – M (24 genes, blue dotted), G (26 CNVs, black dashdotted), and MG (32 genes, 2 CNV, red solid)
