# Supplementary material for: A kernel-based integration of genome-wide data for clinical decision support
Source: Genome Med. 2009 Apr 3;1(4):39. doi: 10.1186/gm39 (PMC2684660; doi:10.1186/gm39)
Supplement: Additional data file 3 — Additional Tables 1-3 show all genes and proteins selected by the best performing models MPT1 for the prediction of WHEELER (25 genes, 12 proteins), pN-STAGE (21 genes, 14 proteins), and CRM (7 genes, 33 proteins) in rectal cancer. Additional Tables 4-7 show, for prostate cancer, the genes and CNVs selected by the best performing models MG for the prediction of GRADE (6 genes, 8 CNVs), STAGE (42 genes, 22 CNVs), METASTASIS (18 genes, 3 CNVs), and RECURRENCE (32 genes, 2 CNVs). All tables additionally show the number of LOO iterations in which each gene, protein, or CNV was selected, their chromosomal region, and whether it is up- or down-regulated. [file gm39-S3.pdf]

Additional file 3 - Genes, proteins, and CNVs selected by the models MPT_1 and MG

Additional lists 1 to 3 show all genes and proteins selected by the best performing models MPT_1 for the prediction of WHEELER (25 genes, 12 proteins), pN-STAGE (21 genes, 14 proteins), and CRM (7 genes, 33 proteins) in rectal cancer. Additional lists 4 to 7 show for prostate cancer the genes and CNVs selected by the best performing models MG for the prediction of GRADE (6 genes, 8 CNVs), STAGE (42 genes, 22 CNVs), METASTASIS (18 genes, 3 CNVs), and RECURRENCE (32 genes, 2 CNVs). All tables additionally show the number of LOO iterations in which each gene, protein, or CNV was selected, their chromosomal region, and whether it is up- or down-regulated.

Additional list 1 - WHEELER: 25 genes and 12 proteins selected by MPT_1

dataset$^\mu$	gene/protein	hits$^\varphi$	region	up/down$^\varsigma$
M	PROK2	36	3p13	up
M	GPR109B	36	12q24.31	up
M	LYZ	36	12q15	up
M	COX-2	36	1q25.2-q25.3	up
M	MNDA	36	1q22	up
M	IL1B	36	2q14	up
M	C5AR1	36	19q13.3-q13.4	up
M	S100A12	36	1q21	up
M	IL8RB	36	2q35	up
M	PAI-2	36	18q21.3	up
M	G0S2	36	1q32.2-q41	up
M	BCL2A1	36	15q24.3	up
M	TNFAIP6	35	2q23.3	up
M	REG1A	34	2p12	up
M	FCGR3B	34	1q23	up
M	TREM1	34	6p21.1	up
M	TMEM71	33	8q24.22	up
M	AQP9	32	15q22.1-q22.2	up
M	CXCL6	31	4q21	up
M	S100A8	29	1q21	up
M	NCF2	29	1q25	up
M	SELE	25	1q22-q25	up
M	REG1B	19	2p12	up
M	KLK11	19	19q13.3-q13.4	down
M	MMP-1	15	11q22.3	up
P	IL-4	36	5q31.1	up
P	ferritin	36	19q13.3-q13.4; 11q13	down
P	IL-6	36	7p21	up
P	apolipoprotein H	36	17q23-qter	down
P	EGF	36	4q25	up
P	MMP-2	36	16q13-q21	up
P	TGFα	36	2p13	down
P	lymphotactin	36	1q23	up
P	erythropoietin	34	7q22	up
P	alpha-2 macroglobulin	33	12p13.3-p12.3	down
P	GM-CSF	24	5q31.1	up
P	insulin	11	11p15.5	up

$^\mu$ M=microarray data; P=proteomics data

$^\varphi$ number of occurrences of the gene/protein in the 36 LOO iterations

$^\varsigma$ up/down-regulation in the good responders w.r.t. moderate or poor responders

Additional list 2 - pN-STAGE: 21 genes and 14 proteins selected by MPT_1

dataset$^\mu$	gene/protein	hits$^\varphi$	region	up/down$^\varsigma$
M	REG4	36	1p13.1-p12	down
M	ITLN1	36	1q22-q23.5	down
M	SI	36	3q25.2-q26.2	down
M	zymogen granule protein 16	36	16p13.3	down
M	SPINK4	36	9p13.3	down
M	MUC2	36	11p15.5	down
M	GCG	36	2q36-q37	down
M	CD177	36	19q13.2	down
M	CA1	36	8q13-q22.1	down
M	CLCA1	36	1p31-p22	down
M	PLA2G2A	36	1p35	down
M	CA2	36	8q22	down
M	CLCA4	36	1p31-p22	down
M	FCGBP	36	19q13.1	down
M	CLDN8	36	21q22.11	down
M	B3GALT5	35	21q22.3	down
M	INSL5	33	1p31.1-p22.3	down
M	CEL	31	9q34.3	up
M	RARRES1	31	3q25.32-q25.33	down
M	CA4	19	17q23	down
M	LOC253012	18	7q21.3	down
P	carcinoembryonic antigen	36	19q13.1-q13.2	down
P	IL-1ra	36	2q14.2	up
P	cancer antigen 19-9	36		down
P	MIP-1beta	36	17q12	up
P	ferritin	36	19q13.3-q13.4; 11q13	down
P	IL-3	36	5q31.1	up
P	IL-1beta	36	2q14	down
P	factor VII	36	13q34	down
P	stem cell factor	36	12q22	down
P	beta-2 microglobulin	36	15q21-q22.2	up
P	alpha-fetoprotein	34	4q11-q13	down
P	thyroxine binding globulin	32	Xq22.2	down
P	IL-8	28	4q13-q21	down
P	TNF RII	24	1p36.3-p36.2	up

$^\mu$ M=microarray data; P=proteomics data

$^\varphi$ number of occurrences of the gene/protein in the 36 LOO iterations

$^\varsigma$ up/down-regulation for no lymph nodes w.r.t. at least one regional lymph node

Additional list 3 - CRM: 7 genes and 33 proteins selected by MPT_1

dataset$^\mu$	gene/protein	hits$^\varphi$	region	up/down$^\varsigma$
M	CYP1B1	35	2p21	down
M	PAI-2	32	18q21.3	down
M	CA12	19	15q22	down
M	IGKV1D-13	18	2p12	up
P	factor VII	36	13q34	up
P	ICAM-1	36	19p13.3-p13.2	up
P	SHBG	36	17p13-p12	up
P	betacellulin	36	4q13-q21	up
P	alpha-1 antitrypsin	36	14q32.1	up
P	C reactive protein	36	1q21-q23	up
P	carcinoembryonic antigen	36	19q13.1-q13.2	down
P	MMP-2	36	16q13-q21	up
P	adiponectin	36	3q27	down
P	thrombospondin-1	36	15q15	up
P	apolipoprotein H	36	17q23-qter	down
P	EGF-R	36	7p12	up
P	SGOT	36	6q14-q15	down
P	IL-18	35	11q22.2-q22.3	down
P	MMP-3	35	11q22.3	down
P	lymphotactin	35	1q23	up
P	tissue factor	35	1p22-p21	down
P	MCP-1	35	17q11.2-q12	up
P	MDC	34	16q13	down
P	prostate specific antigen; free	34	19q13.41	down
P	glutathione S-transferase	34		up
P	IgM	33		up
P	stem cell factor	33	12q22	up
P	EGF	32	4q25	up
P	serum amyloid P	31	1q21-q23	up
P	brain-derived neurotrophic factor	30	11p13	up
P	HB-EGF	28	5q23	up
P	lipoprotein (a)	27	6q26	up
P	IL-4	26	5q31.1	up
P	IL-3	25	5q31.1	up
P	RANTES	22	17q11.2-q12	up
P	apolipoprotein A1	22	11q23-q24	up
P	cancer antigen 19-9	21		down

$^\mu$ M=microarray data; P=proteomics data

$^\varphi$ number of occurrences of the gene/protein in the 36 LOO iterations

$^\varsigma$ up/down-regulation in negative CRM w.r.t. positive CRM

Additional list 4 - GRADE: 6 genes and 8 CNVs selected by *MG*

dataset$^{\mu}$	gene/CNV	hits$^{\varphi}$	region	up/down$^{\varsigma}$
M	SFRP4	55	7p14.1	up
M	PLA2G2A	55	1p35	up
M	VCAN	55	5q14.3	up
M	ALOX15B	36	17p13.1	down
M	COL3A1	33	2q31	up
M	SEMA4G	32	10q24.31	down
G	SMYD5	55	2p13.2	up
G	NBEAL2	55	3p21.31	up
G	GPD1L	55	3p22.3	up
G	MRPL45	54	17q21.2	up
G	HOMER3	54	19p13.11	down
G	FGF13	54	Xq26.3	up
G	KCTD12	51	13q22.3	up
G	EDF1	48	9q34.3	down

$^{\mu}$ M=microarray data; G=genomic data

$^{\varphi}$ number of occurrences of the gene/CNV in the 55 LOO iterations

$^{\varsigma}$ up/down-regulation in high-grade w.r.t. low-grade

Additional list 5 - STAGE: 42 genes and 22 CNVs selected by MG

dataset$^{\mu}$	gene/CNV	hits$^{\varphi}$	region	up/down$^{\varsigma}$
M	MAGEA4	50	Xq28	down
M	PEBP4	50	8p21.3	down
M	CDO1	50	5q22-q23	up
M	VIL1	50	2q35-q36	down
M	TM4SF5	50	17p13.3	down
M	DUSP19	50	2q32.1	down
M	HDAC9	50	7p21.1	up
M	SEMA4G	50	10q24.31	down
M	ANPEP	50	15q25-q26	down
M	POU4F1	50	13q31.1	down
M	XKR4	50	8q12.1	down
M	F5	50	1q23	up
M	AHSG	50	3q27	down
M	SECTM1	50	17q25	down
M	GPRASP1	49	Xq22.1	down
M	WDR72	49	15q21.3	down
M	DNASE2B	48	1p22.3	up
M	CXCL14	48	5q31	up
M	MAGEA8	48	Xq28	down
M	NCALD	48	8q22.2	up
M	C11orf9	48	11q12-q13.1	down
M	CRYGC	48	2q33-q35	down
M	TRAPPC6B	48	14q21.1	down
M	SLAMF8	47	1q23.2	up
M	AGR3	45	7p21.1	up
M	TCTE1	45	6p21.1	down
M	TFF3	43	21q22.3	down
M	GDEP	41	4q21.1	down
M	SLC10A7	41	4q31.22	up
M	C5orf23	40	5p13.3	down
M	ESPNL	39	2q37.3	down
M	DOC2A	38	16p11.2	down
M	MED20	35	6p21.1	down
M	MXRA5	33	Xp22.33	up
M	LOC375646	32	8p23.1	down
M	LOC374495	30	13q13.2	down
M	PHYHIPL	30	10q11	down
M	SLC26A3	30	7q31	down
M	KIAA1244	30	6q23.3	up
M	RHCG	27	15q25	down
M	TREML3	24	6p21.1	down
M	MUC13	24	3q21.2	down

$^{\mu}$ M=microarray data; G=genomic data
$^{\varphi}$ number of occurrences of the gene/CNV in the 50 LOO iterations
$^{\varsigma}$ up/down-regulation in advanced stage w.r.t. early stage

Additional list 5 (cont.) - STAGE: 42 genes and 22 CNVs selected by *MG*

dataset$^{\mu}$	gene/CNV	hits$^{\varphi}$	region	up/down$^{\varsigma}$
G	MCCC1	50	3q27	up
G	OS9	50	12q13	down
G	DCUN1D5	50	11q22.3	up
G	TRO	50	Xp11.22-p11.21	up
G	CCDC101	50	16p11.2	down
G	ALX3	50	1p13.3	down
G	EBNA1BP2	50	1p35-p33	up
G	BCL3	49	19q13.1-q13.2	up
G	GPD1L	49	3p22.3	up
G	NGB	49	14q24.3	down
G	IL31RA	49	5q11.2	down
G	SEPW1	48	19q13.3	up
G	PSTPIP1	48	15q24-q25.1	down
G	RNASEL	48	1q25	up
G	RNF34	45	12q24.31	down
G	ABHD3	41	18q11.2	down
G	SVOPL	41	7q34	up
G	TSEN34	38	19q13.4	up
G	ZNF7	36	8q24	up
G	BHMT	35	5q13.1-q15	down
G	SMYD5	32	2p13.2	up
G	NELF	29	9q34.3	up

$^{\mu}$ M=microarray data; G=genomic data
$^{\varphi}$ number of occurrences of the gene/CNV in the 50 LOO iterations
$^{\varsigma}$ up/down-regulation in advanced stage w.r.t. early stage

Additional list 6 - METASTASIS: 18 genes and 3 CNVs selected by MG

dataset$^{\mu}$	gene/CNV	hits$^{\varphi}$	region	up/down$^{\varsigma}$
M	AHSG	50	3q27	down
M	ERG	50	21q22.3	up
M	PROM2	50	2q11.1	up
M	LOC375475	50	5q33.1	up
M	TGM4	50	3p22-p21.33	down
M	SNF1LK	50	21q22.3	down
M	LOC283588	50	14q32.11	up
M	ARL17P1	50	17q21.32	up
M	AREG	49	4q13-q21	down
M	VAV3	49	1p13.3	up
M	TLR8	47	Xp22	up
M	FAM19A2	46	12q14.1	up
M	PCDH18	46	4q31	up
M	RAMP3	45	7p13-p12	up
M	NDRG3	45	20q11.21-q11.23	up
M	CYP4F8	43	19p13.1	up
M	SLCO2B1	37	11q13	up
M	ADAMTS1	26	21q21.2	down
G	SYTL3	50	6q25.3	down
G	NPR2	48	9p21-p12	down
G	TRO	43	Xp11.22-p11.21	up

$^{\mu}$ M=microarray data; G=genomic data
$^{\varphi}$ number of occurrences of the gene/CNV in the 50 LOO iterations
$^{\varsigma}$ up/down-regulation in metastasis w.r.t. no metastasis

Additional list 7 - RECURRENCE: 32 genes and 2 CNVs selected by *MG*

dataset$^{\mu}$	gene/CNV	hits$^{\varphi}$	region	up/down$^{\varsigma}$
M	AZGP1	29	7q22.1	down
M	LIF	29	22q12.2	down
M	FAM149A	29	4q35.2	up
M	NRP1	29	10p12	up
M	TIAM1	29	21q22.1	up
M	FGG	28	4q28	down
M	SEMA4G	27	10q24.31	down
M	BASP1	27	5p15.1-p14	down
M	INPP1	27	2q32	up
M	ATF3	26	1q32.3	down
M	F5	26	1q23	up
M	TRAPPC9	26	8q24.3	up
M	JAG1	26	20p12.1-p11.23	up
M	SPRR1B	25	1q21-q22	up
M	PCYT1B	25	Xp22.11	up
M	KCNG	25	20q13	up
M	C13orf3	24	13q12.11	up
M	SNAP91	23	6q14.2	up
M	C6orf50	23	6p25.1	up
M	RS1	23	Xp22.2-p22.1	up
M	MRAP2	23	6q14.3	up
M	ANKRD29	21	18q11.2	up
M	PEX5L	19	3q26.33	up
M	LAMA3	18	18q11.2	up
M	ANPEP	16	15q25-q26	down
M	SAA1	15	11p15.1	down
M	ERG	14	21q22.3	up
M	ZNF385B	14	2q31.2-q31.3	up
M	ALOX15B	14	17p13.1	down
M	TMEM45B	14	11q24.3	up
M	FICD	14	12q24.1	up
G	KCTD12	27	13q22.3	up
G	SPR	10	2p14-p12	down

$^{\mu}$ M=microarray data; G=genomic data
$^{\varphi}$ number of occurrences of the gene/CNV in the 29 LOO iterations
$^{\varsigma}$ up/down-regulation in recurrence w.r.t. no recurrence
